# Research Trends of Follow-Up Care after Neonatal Intensive Care Unit Graduation for Children Born Preterm: A Scoping Review

**DOI:** 10.3390/ijerph18063268

**Published:** 2021-03-22

**Authors:** So Ra Kang, Haeryun Cho

**Affiliations:** 1 College of Nursing, Ewha Womans University, Seoul 03760, Korea; solarsolar81@naver.com; 2 Department of Nursing, Wonkwang University, Iksan 54538, Korea

**Keywords:** children born preterm, follow-up care, NICU graduation, scoping review

## Abstract

The purpose of this study was to describe the trends of research on follow-up care after neonatal intensive care unit (NICU) graduation for children born preterm. This scoping review was conducted according to Arksey and O’Malley’s guidelines. Reviewed studies were searched in PubMed, CHINAHL, and Web of Science. Fifteen studies were analyzed according to general characteristics, elements of follow-up care after NICU graduation, and characteristics of follow-up care intervention after NICU graduation. Most research was conducted in the medical field (60%), with experimental studies (40%) being the majority, and a few studies focused on families (3%) and parents (3%). The major follow-up care after NICU graduation elements were growth/developmental monitoring and support, continuity of care, parent- and family-centered elements, and a multidisciplinary approach. The intervention methods included home visits, phone calls, video calls, and applications. In addition, the intervention period ranged from two weeks to three years. It is suggested that multidisciplinary research with interactive media for a various age of children over longer periods for further study.

## 1. Introduction

Advances in perinatal and neonatal care have led to improved survival in preterm infants [1,2,3]. The survival rate is approximately 50% in infants aged 24 weeks, but it is 80% to 90% in infants aged 28 weeks and 95% in those aged 32 weeks in the United States [4]. However, preterm birth also increases the risk of chronic diseases and developmental delays that may persist into adulthood and, consequently, require higher levels of health care [5]. Overall, encouraging the growth and development of surviving premature infants is crucial, along with efforts to increase survival rates. 

Premature infants are a high-risk group for delayed growth and neurodevelopmental disorders, and some have neurological sequelae, such as delayed neurodevelopment, decreased intelligence, and cerebral palsy [6,7]. Factors affecting the growth and neurodevelopment of premature infants include birth weight, gestational age, Apgar score, cardiopulmonary resuscitation (CPR) at birth, breastfeeding, ventilation, or length of hospitalization, among others [8,9]. Growth and development interact not only with the biological and intrinsic elements of the child but also with the environment surrounding the child and changes continuously [10]. In particular, the development of infancy has a significant influence on the interaction with the main caregivers as an external environment [11,12,13]. Therefore, there is a need for follow-up care for infants who have graduated from the neonatal intensive care unit (NICU).

Parents of infants born prematurely face the challenging burden of taking adequate care of their children as well as acquiring an appropriate role as a parent after being discharged from the NICU. Particularly, transitioning from hospital to home for parents is a precarious experience. Prior research has reported that they experience increased levels of stress and express feelings of incompetence, such as anxiety and concern about the discharge of their infants from the NICU [14,15]. Parents of infants born preterm sometimes fall into depression due to difficulties in the process of becoming accustomed to the formation of attachment and the peculiar care of infants after NICU graduation [16]. Therefore, parents whose children are discharged from the NICU also have a need for follow-up care. 

Currently, studies on preterm infants discharged from the NICU have been conducted to assess morbidity [17,18], impact of environmental exposures [19], the prevalence of breast feeding [20], and specific therapy for development [21,22]. Studies have also measured the effectiveness of preliminary discharge education in the NICU [23,24]. In addition, there were studies focused on how to increase the participation rate by revealing factors that affect compliance with follow-up care [25,26]. Previous studies had limitations in assessing simple outcomes of growth and development or complications in infants, dealing with aspects of specific topics, or simply measuring post-discharge education. Despite the demands of parents and children who graduated from the NICU, mapping for follow-up care is difficult because no comprehensive research has been conducted.

The scoping review method maps the concepts of a wide range of research areas that have not yet been integrated and identifies differences in the current research areas [27,28]. In addition, it is a research methodology that has more recently attracted attention as a useful method for providing the basic guidelines and a framework for conducting systematic reviews in the future and for suggesting research directions [29,30]. Unlike systematic review or meta-analysis, this method easily identifies scope and character because it is not limited by criteria such as variables and experimental studies and does not require evaluation of research quality [31]. Therefore, we consider it an appropriate research design approach for conducting literature review to understand the latest new research trends related to follow-up care after NICU graduation for children born preterm.

The aim of this study is to identify the trends within five years of research on follow-up care after NICU graduation for children born preterm, and to suggest directions for future research and interventions related to care for patients and their families after discharge from the NICU. The specific objectives of this study were to assess (1) general characteristics of the reviewed studies, (2) the elements of follow-up care after NICU graduation, and (3) the characteristics of the follow-up care interventions.

## 2. Materials and Methods

### 2.1. Research Design

This study was designed for a scoping review proposed by Arksy and O’Malley [27]. This study followed the six stages of scoping review among the literature review methods. This method is known to be useful in identifying the overall trend of research and deriving research topics and aims to discover various research types, key concepts, and supporting resources. 

### 2.2. Research Procedure

#### 2.2.1. Identifying the Research Question

Research questions for establishing search strategies in the scoping review method are recommended to be broadly set to understand a wide range of research areas [27]. Thus, the research question was set as “What is the trend of research on follow-up care after NICU graduation for children born preterm within five years, and what is the direction of future research based on this trend?”.

#### 2.2.2. Identifying Relevant Studies

##### Searching Strategies

This study targets research papers published between January 2016 and December 2020, among research papers related to follow-up care of preterm infants graduating from the NICU. We performed literature searches of the electronic databases (PubMed, Cumulative Index of Nursing and Allied Health Literature, and Web of Science). ‘Premature’, ‘Preterm infant’, ‘NICU’, and ‘Follow-up’ were set as the main search keywords, and two researchers worked together to increase the accuracy and reliability of the search word setting and search process. The search was performed using major indexes for each database (e.g., MeSH term, CINAHL subject headings, etc.) and natural language and Boolean operators; the search terms and search results for each database are shown in Supplementary S1.

##### Searching Result

**Figure 1:**
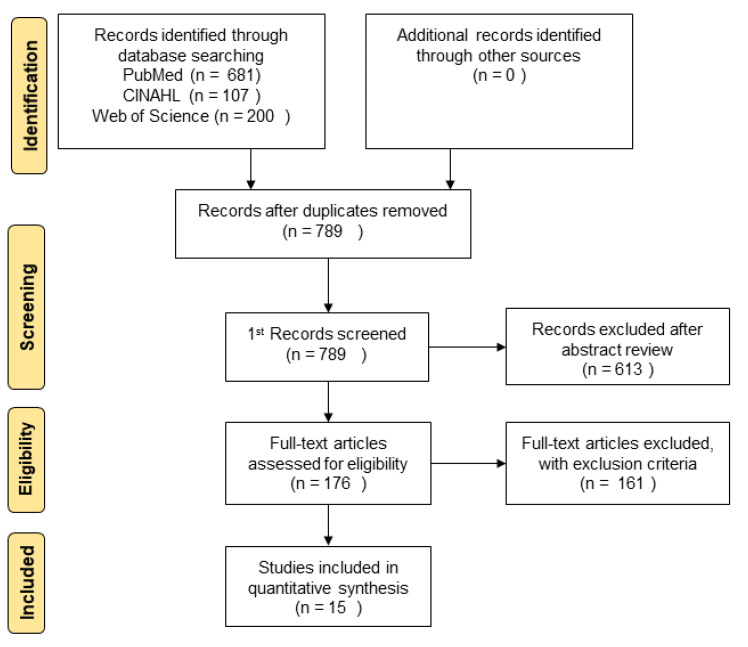
Flow diagram for study selection.

As a result of the search on 3 February 2021, a total of 988 papers were found. 199 duplicate documents were removed using Endnote 20 (Clarivate, Philadelphia, PA, USA); a total of 789 studies were confirmed (Figure 1).

#### 2.2.3. Study Selection

To select the final studies, we conducted a total of three-step reviews. The first step was to review the title and abstract, then review the text, and then to select literature and record data. In every review process, two researchers independently reviewed each other to eliminate selection bias. Studies that did not agree with the results of the review were included and excluded from the final study after reading the original full-text and deciding whether to select a study through an in-depth discussion. Two researchers discussed selecting and agreeing on the criteria for inclusion and exclusion of research that encompasses research questions. 

The inclusion criteria were as follows: (1) studies that focused on the follow-up care of premature infants after graduation from the NICU, (2) original articles published after peer review, and (3) articles written in English. The exclusion criteria were as follows: (1) research limited to the management of specific diseases or treatment, therapy, and (2) editorial, letter to an editor, or conference proceedings.

As a result of the first review (title and abstract), 613 papers were excluded from the 789 papers. The second step was conducted on 176 papers, including 14 papers in which the opinions of the two researchers did not agree. In the second step of the review, full-text documents were secured and reviewed, including documents whose titles were only reviewed because no abstract was provided, and documents wherein the bibliographic information was not clear. As a result of the second step review, a total of 161 papers were excluded, of which 30 contained partial mentions of follow-up care after graduation, 117 studies were unrelated to NICU follow-up care after graduation, five cases involved the completion of discharge education before NICU graduation, six only measurements were later made after discharge, and three were non-English studies. Accordingly, the final 15 studies that focused on follow-up care of premature infants after graduation from the NICU, were written in English language, and were peer-reviewed published literature, were selected (Supplementary S2). Subsequently, a third-step review was conducted in stage four (charting the data). A flow diagram of the data collection, selection, and extraction processes is shown in Figure 1. The scoping review suggested that the quality of selected studies should not be evaluated in order to minimize resource limitations [27,31]. According to this guideline, quality assessment was not conducted in this study. 

#### 2.2.4. Charting the Data

The fourth stage was to classify the collected data and describe them as data, sufficiently review the subject of analysis, and capture important information as a chart. It is recommended to use Microsoft Excel (Microsoft Inc., Redmond, WA, USA) to fill in the information of the study [27]. Previously, the searched documents were managed using Endnote 20 (Clarivate, Philadelphia, PA, USA), a bibliographic management software, and the main results were analyzed and recorded in Excel (Microsoft Inc., Redmond, WA, USA). Recording data is the process of updating the data entry format in the iterative process of extracting data from the paper. For the record format, we applied the category suggested by Arksey & O’Malley [27]. We recorded and analyzed the author, publication year, publishing country (region), subject and purpose of research, research method (design), results regarding measurement (variables and tools), and major key research results. Two researchers independently analyzed and filled out the contents of the literature in the final recorded form, and then exchanged opinions on the recorded data through regular research meetings (Supplementary S3). After data were recorded in the Excel file, the frequency and percentage were presented using Microsoft Excel (Microsoft Inc., Redmond, WA, USA).

#### 2.2.5. Collating, Summarizing, and Reporting Results

The scoping review takes an overview of all data as important, and unlike a systematic review, it is not necessary to assess the quality of the evidence because it is not intended to draw a generalized conclusion or only partially included or excluded [27]. Therefore, the researchers selected and recorded the study according to the method guidelines of the scoping review, and synthesized and summarized the study in the final step.

#### 2.2.6. Consultation Exercise

This stage is optional and is a process of informing stakeholders outside the research team about the results of the subject scope literature review and seeking insights from them to secure the validity of the results. At this stage, clinical practitioners can become advisory groups [27]. Therefore, we consulted about the derived results with two NICU staff nurses, with more than 20 years of experience, and one pediatric nursing professor. According to their advice, the results of this study were modified. For instance, researchers considered “tracking immunization status” as a developmental task and classified it as “developmental monitoring”, but nurses working on the ward had the view that vaccinations of patients and monitoring were also being performed in the ward, and this should be viewed as a “continuity of care from the NICU” In addition, “learning caregiving and parenting” was first categorized as “parent care”, however, it was reclassified as “parent support” according to the advice of the nursing professor that it was support rather than care.

## 3. Results

### 3.1. General Characteristics of Reviewed Studies

A total of 988 articles were searched and 973 articles were excluded (199 duplicate, 774 title, abstract and full-text review), and the results of finally analyzing 15 literatures are as follows. Table 1 lists the general characteristics of the reviewed studies. In the published year, 2018 was the largest (40.0%). In the academic field, medicine was the most common (60.0%), followed by nursing (20.0%). The USA was the most common (73.3%) country where the study was conducted, followed by Sweden (13.3%). As for the study design, experimental studies were the most common (40.0%), followed by descriptive research studies (33.3%) and review studies (26.7%). As for the study subjects, infants born prematurely were the most common (40.0%), followed by families and parents of them (13.3%). In addition, 6.7% of toddlers were born preterm.

### 3.2. Elements of Follow-Up Care after NICU Graduation

Table 2 lists the elements of follow-up care after NICU graduation. Four papers (R4, R8, R11, R13) showed continuously monitoring the growth and development of children born preterm or high-risk newborns as follow-up care after NICU graduation. In addition, paper numbers 2, 8, 6 and 11 dealt with the support for infants who were treated in the NICU not only for physical growth but also exercise, cognition, and social development. In papers R3, R6 and R11, efforts to meet children’s feeding and nutritional needs were included in follow-up care after NICU graduation. 

Supporting baby care for parents to raise their children well at home after discharge was considered as follow-up care after NICU graduation in four papers (R1, R8, R11, R13). Three papers (R1, R8, R13) were caring for parents raising their children discharged from the NICU, including parental roles, psychological support, and parental mental health. In addition, attachment between parents and children was found in two papers (R1, R11), and family-centered care (FCC) was found in two papers (R3, R10).

In three papers (R4, R7, R11), follow-up care after NICU graduation included continuous therapeutic management if the care performed by the NICU was necessary even after discharge. In addition, there were five papers (R4, R6, R8, R10, R12) that considered multidisciplinary teams including doctors, nurses, occupational therapists, and social workers as providers of follow-up care after NICU graduation. Home nursing services were found to be an element of follow-up care. It was also found that tending appropriately to the various needs of children and parents was an element of follow-up care.

### 3.3. Characteristics of Follow-Up Care Intervention after NICU Graduation

Table 3 lists the characteristics of the follow-up care intervention after NICU graduation. Eight studies dealt with follow-up care interventions. 

**Figure 2:**
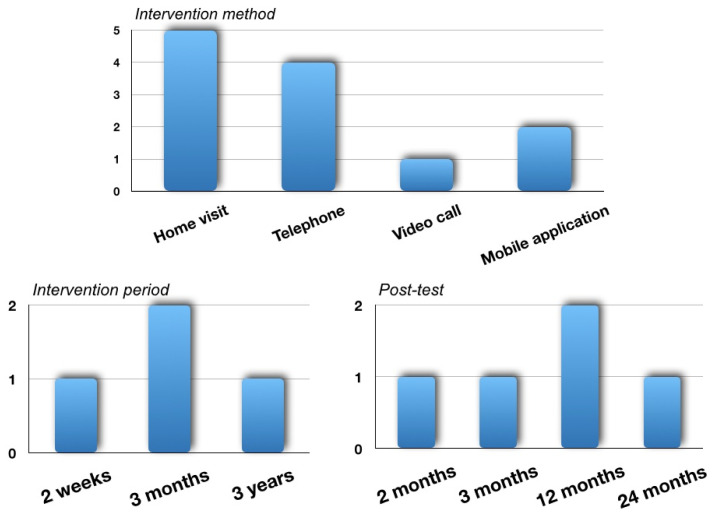
Classification for intervention of follow-up care after NICU graduation.

In terms of intervention methods, there were five papers (R2, R10, R12, R14, R15) that performed home visits, and five papers (R3, R10, R13, R14, R15) that conducted support by phone including a video call. There were two papers (R11, R13) that utilized mobile application. The duration of the intervention varied from 2 weeks (R3) to 3 months (R10) and 3 years (R15). Post-tests were performed to verify the long-term effects from 2 months (R3) or 3 months (R15) after discharge from the NICU to 12 months of corrective age (R2, R11) and 2 years after birth (R10) (Figure 2). 

Outcome variables and measurement tools varied according to the aim of the study and the goals of the intervention. Four papers (R10, R12, R13, R15) assessed readmissions or emergency room visits. One paper (R11) measured children’s physical growth, and two papers (R2, R11) assessed children’ s developmental status using the Bayley Scale of Infant and Toddler Development—III Edition. Three papers (R3, R11, R14) evaluated parent-child interaction and attachment. In addition, parenting stress, quality of life, medical costs, and knowledge were measured.

In terms of the contents of the intervention, there were three papers (R10, R11, R15) wherein follow-up intervention was provided by the multidisciplinary team and home visits were provided by neonatal nurse practitioner (NNP) mainly. Phone support was provided by health professionals, but parents could connect the phone whenever they wanted (R10, R13, R15). In addition, two-way interactions using home visits, telephone support, mobile applications, and video calls were attempted (R10, R13, R14, R15).

## 4. Discussion

This study aimed to investigate the research trends in follow-up care after NICU graduation. To this end, 15 studies published within the last five years were analyzed using scoping reviews.

Of the studies used in the analysis, 60% were medical articles in present study. Descriptive studies using cohort data or studies discussing the design of follow-up care have been conducted in medical papers. As the survival rate of high-risk newborns increases, owing to advances in medical technology and social policy [1,2,3], there has been an emerging need to manage complications after discharge from the NICU or continuous follow-up for appropriate growth development [1,32,33]. In this context, the most common and easily accessible healthcare system for newborns and their families discharged from the NICU is the primary clinic [25,34,35]. The fact that there were many medical studies primarily providing follow-up care affords the worthwhile interpretation, in the context of this study, that interest in follow-up care and research is in its nascent stages. In other words, it can be inferred that follow-up care is a blue ocean that requires active research with interest in local communities and various academic fields.

In this study, it was found that forming a multidisciplinary team including doctors, nurses, occupational therapists, and social workers is important for follow-up care. Park et al. [15] suggested that a team of multidisciplinary experts should provide individual interventions for children born prematurely, as their parents have overall needs, including medical complications, delayed growth and development, and general handling of newborns. In the studies by Feehan et al. [36] and Liu et al. [37], a multidisciplinary team was formed for follow-up care after discharge from the NICU. In this study, there were studies that formed such a multidisciplinary team, but many studies showed that experts in each field of medicine, nursing, physical therapy, and occupational therapy performed individual interventions. In accordance with the demand that multidisciplinary follow-up care be emphasized, efforts to establish each role and responsibility for each field of experts are needed in the future.

As the key elements of follow-up care, support of parenting and parents, as well as care related to the child’s growth and development, was included. According to Park et al. [15], who analyzed social network services (SNS) using text mining to understand the support needs of parents caring for their children who have been discharged from the NICU, parents raising children born as preterm infants have difficulties in handling and raising babies and experience anxiety and concerns regarding their children’s physical and developmental health. It has been reported that parents who care for children born to preterm infants have less parental role acquisition and higher parenting stress than parents who raise full-term infants [38,39,40]. In this respect, it is similar to this study in that Park et al. [15] emphasized the necessity of intervention, including general infant care, as well as specific care for prematurity, to enhance the parenting efficacy of preterm infants. In addition, parental behavior affects children’s optimal growth and development [12,13]. Thus, providing general infant care along with interventions for parents’ concerns and difficulties is an important element to be included in follow-up care.

The philosophical basis of child health nursing care lies in FCC, which considers both children and their families as targets for nursing care [41,42]. Family-centeredness is an important concept not only in nursing but also in medicine [36]. This philosophy was also reflected in the results of this study, in which attachment to the FCC was confirmed as an element of follow-up care. Having a healthy relationship among preterm infants, parents, and siblings at home after NICU discharge is crucial for the growth and development of children [12,13,14,43]. Relationships between family members, as well as partnerships with families of children discharged from the NICU and medical staff, are important concepts for the health of children [44]. Therefore, the results of this study are meaningful and prove that it is important to establish a positive attachment relationship between parents and children and support the optimal growth and development of premature children through family cooperation as an element of follow-up care. Although family and parents play important roles in follow-up care, this study found that 13.3% of each of these studies were published. Therefore, follow-up care research based on FCC should be conducted more proactively in the future.

The results of this study confirmed that home visits, on-call phones, video phones, and mobile applications were used as intervention methods for follow-up care after NICU graduation. The common point is that a follow-up care service-centered on patients rather than provider-centered care is possible. Because of the limitations in human and financial resources and difficulties in health service accessibility, it is not easy to provide health-related interventions for children born prematurely and their families [45,46]. Particularly, in the coronavirus disease (COVID)-19 pandemic, non-face-to-face intervention has become necessary for the continuation of health services for a medically vulnerable group such as children born as premature infants and their families. A web-based intervention such as a videophone and mobile application is a method that can be expected to have a lower cost and greater impact while being a non-face-to-face intervention [46]. Therefore, various media should be developed to enable interaction between children and their families and health professionals in web-based interventions for effective follow-up care after NICU graduation.

The follow-up care interventions after NICU graduation were found to vary from two weeks to three years in this study. Previous studies have reported that children born prematurely are at a risk of developmental delays over longer periods of time, including infants, toddlers, preschoolers, and school-goers [11,47]. In other words, follow-up care after NICU graduation needs to cover the entire childhood period until the child’s growth and development is complete. Although a study analyzing this was conducted on preterm toddlers, it was confirmed that the study was limited mainly to infancy; thus, further study should be conducted on children of a wider developmental stage. Each child’s developmental stage has different main tasks and developmental goals [12]. Therefore, it is necessary to develop the content of follow-up care suitable for the developmental needs of children at each developmental stage.

This study was meaningful in that it was able to grasp the trend of the research and suggest directions for future research at a time when interest in follow-up care after NICU graduation is increasing. However, the limitation is that there may be a bias in the selection of the target paper, limited to studies within five years, and studies published in English. Based on this study, we look forward to active research and attempts at conducting more diverse follow-up care after NICU graduation.

## 5. Conclusions

This study was conducted to identify the research trend of follow-up care after NICU graduation, and to provide basic data to seek and activate the research direction of follow-up care after NICU graduation. The main results of analyzing 15 studies published within the last five years according to the stage of scoping review analysis are as follows. Research conducted so far has been conducted in the medical science field and interventions by multidisciplinary teams are being attempted but are still in the early stages. Recent studies reflecting the family-centered issue of child nursing care and pediatric treatment have not been sufficient. According to the analysis, children and their families discharged from the NICU need to use media that can provide customized interventions. In addition, although follow-up care is required for a long period of time, it can be found that studies were mainly conducted in infancy after discharge. 

Further research and intervention suggestions are as follows: First, the multidisciplinary approach of follow-up care after NICU graduation should be actively attempted in research and clinical fields. The outcomes of these efforts will ultimately serve as the basis for developing guidelines for a multidisciplinary team of follow-up care after NICU graduation. Second, the factors identified in this study as well as the needs of various children’s developmental stages (infancy, preschool age, school age, and adolescence) should be reflected in providing follow-up care after NICU graduation in the future. Third, it is necessary to use a variety of media capable of non-face-to-face and interactive interactions targeting children and families discharged from the NICU in accordance with the current needs.

## Figures and Tables

**Table 1:** General characteristics of reviewed studies (n = 15).

Category	Characteristics	Total
		n (%)
Publish year	2016	2 (13.3)
	2017	2 (13.3)
	2018	6 (40.0)
	2019	3 (20.0)
	2020	2 (13.3)
Academic field	Medicine	9 (60.0)
	Nursing	3 (20.0)
	Others	2 (13.3)
	Unknown	1 (6.7)
Research nation	USA	11 (73.3)
	Sweden	2 (13.3)
	Spain	1 (6.7)
	India	1 (6.7)
Study design	Review	4 (26.7)
	Experimental study	6 (40.0)
	Descriptive study	5 (33.3)
Subject	Infant born preterm	6 (40.0)
	Family	2 (13.3)
	Parents	2 (13.3)
	Toddler born preterm	1 (6.7)
	Paper	1 (6.7)
	Not applicable	3 (20.0)

USA = United State of America.

**Table 2:** Elements of follow-up care after neonatal intensive care unit (NICU) graduation.

Element	Contents (Number of Reviewed Paper)
Developmental monitoring	◆ Routine developmental surveillance [R4] ◆ Developmental monitoring [R8] ◆ Remote monitoring of preterms growth parameters, developmental milestones [R11] ◆ Check of weight, length, and head circumference [R13]
Growth and developmental support	◆ Motor development [R2] ◆ Cognitive development [R2] ◆ Catch-up growth [R6] ◆ Gross/fine motor, cognitive/linguistic, behavior/social interaction [R8] ◆ Stimulating activities and toy safety [R11]
Feeding & nutrition	◆ Support for breastfeeding [R3] ◆ Feeding, breastfeeding [R6] ◆ Nutrition: achieving energy, protein, and mineral needs [R6] ◆ Monitoring the breastfeeding [R11] ◆ Complementary feeding practice [R11]
Parenting support	◆ Learning caregiving and parenting [R1] ◆ Home carryover activities [R8] ◆ Hygiene practices including bath, massage and diaper care [R11] ◆ Facilitating early recognition of danger signs [R11] ◆ How to care infant [R13]
Parent care	◆ Parental role development [R1] ◆ Psychological consequences of a preterm birth and infant hospitalization [R1] ◆ Need for social and professional supports which appear to reflect parental challenges [R1] ◆ Parents’ mental health [R8] ◆ Parents’ questions and concerns [R13]
Attachment	◆ Development of parent-infant relationships [R1] ◆ Parent-infant interaction [R11]
Family centered care	◆ Person centered care [R3] ◆ Individualized family-centered [R10]
Continuity of care from the NICU	◆ Continuity of care from the NICU to primary care [R4] ◆ Technology dependence: oxygen, apnea monitor, tracheostomy and home ventilator, feeding tube [R6] ◆ Prescribed medication [R6] ◆ Tracking immunization status [R11]
Multidisciplinary team	◆ Care coordination [R4] ◆ Subspecialty clinic referrals: neurologic problems (malformations of the CNS, ischemic brain injury, hemorrhagic brain injury, other neurologic problems), muscle tone abnormalities, CP, sensory impairment (vision, hearing), developmental delay [R6] ◆ Referrals for necessary therapeutic interventions [R8] ◆ An interdisciplinary team of physicians, nurse practitioners, social workers, and family resource specialists [R10] ◆ Occupational therapy [R12]
Home nursing	◆ Home nursing service [R6]
Support for various needs	◆ Proactive screening to address medical and social needs [R4] ◆ Financial resources [R8] ◆ Culturally sensitive support [R10] ◆ Link the family to appropriate community resources [R10]

CNS = central nervous system; CP = cerebral palsy; NICU = neonatal intensive care unit.

**Table 3:** Characteristics of follow-up care after NICU graduation.

No.	Intervention Name	Intervention Goal	Intervention Method	Experimental Period	Intervention Contents (Provider)	Post Test	Outcome (Scale)
R2	Supporting play exploration and early development intervention (SPEEDIE)	- To provide an enriched environment and increased opportunities for infant initiated movements during the first months of life in order to enhance the infant’s development during and after the intervention period.	- Phase 1: coaching (video clip or dolls) - Phase 2: visiting, developmental play, booklet	- Phase 1 (NICU): 3 weeks - Phase 2 (home): 12 weeks	(Phase 1) - Response to the infant’s behavioral cues based on the synactive theory of development - Positive and negative interaction available to parents - Behavioral states, self-calming, environmental modification, and choosing times for feeding and play based interactions - Identifying cues to stop, alter, or delay interactions during caregiving, feeding, play activities (Phase 2) - Motor and cognitive developmental play activities	- Follow up 1: 1 month after end phase 2 - Follow up 2: 3 months after end phase 2 - Follow up 3: 12 months CA	- Reaching skill - Problem-solving behaviors (Early Problem Solving Indicator) - Neuromotor control and development (Test of Infant Motor Performance, Bayley III)
R3	Proactive telephone support	Not reported	- Daily telephone call to mother (reactive telephone support)	- 14 days after discharge including weekends	- Information about breastfeeding support - Discussions about breastfeeding support	- 8 weeks after discharge	- Exclusive breastfeeding - Breastfeeding evaluation (Maternal Breastfeeding Evaluation Scale) - Attachment (Maternal Post-natal Attachment Scale) - Parenting stress (Swedish Parenting Stress Questionnaire) - Quality of life (SF-36)
R10	Transition Home Plus program	To provide a continuum of individualized family-centered, culturally sensitive support, provide education, and link the family to appropriate community resources	- Call - Home visit	90 days	- Call within 48 h (Social worker or family resource specialist) - Findings of all visits communicated with primary care physician (MD, NNP, social worker, family resource specialist) - 24/7 on call (MD or NNP) - Home visit for infant/family assessment (NNP) - Calls to and from family and PCP as needed (MD, NNP, social worker, family resource specialist) - Edinburgh at 30 days; facilitate referrals as needed (Social worker or family resource specialist) - 1- and 3-month clinic assessment (MD, NNP, social worker, family resource specialist)	Eight 3-month quarters after infant’s birth	- Total Medicaid spending - Emergency department visit - Readmission
R11	mHealthPHCP	Empowering mothers of preterms and the community health workers in the care of preterms at home after discharge from the NICU	Mobile application	Not report	- Remote monitoring of preterms growth parameters - Developmental milestones - Health status after discharge from hospital - Track immunization status - Scheduling postnatal home visits for ASHAs - Facilitating early recognition of danger signs and prompt referral by ASHAs - Provide direct access to health information on breastfeeding - Expression of breast milk and feeding - Kangaroo mother care - Hygiene practices including bath - Massage and diaper care - How t o monitor baby’s growth - Complementary feeding - Stimulating activities and toy safety - Identification and management of danger signs	3, 6, 9, and 12 months of CA	- Parent-infant interaction (LoTTS parent–infant interaction coding system) - Quality of parent-infant interaction (Global Rating Scale) - Growth of preterm (Calibrated instrument) - Development of preterm (Bayley III) - Compliance to mHealthPHCP (Compliance Rating Scale)
R12	Baby Bridge program	To minimize the gaps in therapy services that high risk premature infants often experience after discharge from the NICU	Home visit	Not report	Not report	Not report	- Number of visits - Total payments - Total expenses of the Baby Bridge program
R13	Telemedicine	Not report	- Web application - Video call (skype) - Response to the parents’ conventional telephone calls	Not report	- Infant’s general health - Activity level - Sleeping pattern - Nutrition (including tube feeding - Spitting up - Skin-to-skin care - Baby’s weight and length data in graphic form - Internal messages	Not applicable	- Satisfaction with the use of telemedicine - Need for scheduled - Number of Emergency visit and hospitalization
R14	Early Discharge Programme	To advance discharge and prevent the newborn from suffering from the complications of a prolonged hospitalization	- Home visit - Telephone assistance	Not report	Not report	Not applicable	- Knowledge of infant care - Knowledge of breast-feeding - Knowledge of health resources - Parent-infant attachment - Social support - Psychosocial adjustment: life change
R15	Transition home program	Not report	- Home visit - Call	3 years	- Post discharge call within 24 h (clinical social worker or family resource specialists) - NNP visit within 1 week for assessment, management and support - Standard Visiting Nurse visits - Discharge summary to PCP - Referral to early intervention - 24/7 on call by study physicians for 90 days post discharge (MD and NPs) - Real-time alerts to staff of ER visits and hospitalizations from state Current Care - secure database (MD and NPs) - Seen in Clinic at 1 and 3 months (MD and NPs) - Edinburgh administered at 1 month post discharge (clinical social worker or family resource specialists) - Phone communication at 1 and 3 months (clinical social worker or family resource specialists)	- 1 week post discharge - 30 days post discharge - 90 days post discharge	- ER visit - Hospital admissions

ASHAs = Accredited Social Health Activists; Bayley III = Bayley Scale of Infant and Toddler Development—III Edition; CA = corrected age; ER = emergency room; MD = medical doctor; NICU = neonatal intensive care unit; NNP = neonatal nurse practitioner; NP = nurse practitioner; PCP = primary care provider; SF-36 = Short Form Health Survey—36.

## Data Availability

Data is contained within the supplementary material.

## Supplementary Materials

The following are available online at https://www.mdpi.com/1660-4601/18/6/3268/s1, Supplementary S1, Database search strategies—Search Formula. Supplementary S2. A List of the Literature Reviewed for the study. Supplementary S3, Summary of the Reviewed Researches.

## References

[1] Altimier L., Phillips R. (2016). The neonatal integrative developmental care model: Advanced clinical application of the seven core measures for neuroprotective family-centered developmental care. Newborn Infant Nurs. Rev..

[2] Chung Y.S. (2016). Future of neonatology in Korea; the way forward. J. Korean Med. Assoc..

[3] Mohammed R.E., Khamis G.M., Sabry Y.Y. (2018). Effect of preterm neonates’ developmental supportive care program on nurses’ performance. Nurs. Health Sci..

[4] University of Utah Health Health Outcomes for Preemies Salt Lake City: University of Utah Health. https://healthcare.utah.edu/womenshealth/pregnancy-birth/preterm-birth/when-is-it-safe-to-deliver.php.

[5] Synnes A., Hicks M. (2018). Neurodevelopmental outcomes of preterm children at school age and beyond. Clin. Perinatol..

[6] Amer R., Moddemann D., Seshia M., Alvaro R., Synnes A., Lee K.S., Shah P.S. (2018). Neurodevelopmental outcomes of infants born at <29 weeks of gestation admitted to canadian neonatal intensive care units based on location of birth. J. Pediatr..

[7] Do C.H.T., Kruse A.Y., Wills B., Sabanathan S., Clapham H., Pedersen F.K., Pham T.N., Vu P.M., Børresen M.L. (2020). Neurodevelopment at 2 years corrected age among Vietnamese preterm infants. Arch. Dis. Child..

[8] Holm K.G., Clemensen J., Brødsgaard A., Smith A.C., Maastrup R., Zachariassen G. (2019). Growth and breastfeeding of preterm infants receiving neonatal tele-homecare compared to hospital-based care. J. Neonatal Perinat. Med..

[9] Johnson S., Evans T.A., Draper E.S., Field D.J., Manktelow B.N., Marlow N., Mattews R., Petrou S., Seaton S.E., Smith L.K. (2015). Neurodevelopmental outcomes following late and moderate prematurity: A population-based cohort study. Arch. Dis. Child Fetal Neonatal Ed..

[10] Fumagalli M., Provenzi L., De Carli P., Dessimone F., Sirgiovanni I., Giorda R., Cinnante C., Squarcina L., Pozzoli U., Triulzi F. (2018). From early stress to 12-month development in very preterm infants: Preliminary findings on epigenetic mechanisms and brain growth. PLoS ONE.

[11] Charkaluk M.L., Truffert P., Marchand-Martin L., Mur S., Kaminski M., Ancel P.Y., Pierrat V., Epipage Study Group (2011). Very preterm children free of disability or delay at age 2: Predictors of schooling at age 8: A population-based longitudinal study. Early Hum. Dev..

[12] Oh J.A., Kim Y.Y., Park Y.K., Yoo S.Y., Im M.H., Cho H. (2020). Human Growth and Development.

[13] Treyvaud K., Doyle L.W., Lee K.J., Ure A., Inder T.E., Hunt R.W., Anderson P.J. (2016). Parenting behavior at 2 years predicts school-age performance at 7 years in very preterm children. J. Child Psychol. Psychiatry.

[14] Granero-Molina J., Fernández Medina I.M., Fernández-Sola C., Hernández-Padilla J.M., Jiménez Lasserrotte M.D.M., López Rodríguez M.D.M. (2019). Experiences of mothers of extremely preterm infants after hospital discharge. J. Pediatr. Nurs..

[15] Park J.H., Lee H., Cho H. (2021). Analysis of the supportive care needs of the parents of preterm children in South Korea using big data text-mining: Topic modeling. Child Health Nurs. Res..

[16] Leahy-Warren P., Coleman C., Bradley R., Mulcahy H. (2020). The experiences of mothers with preterm infants within the first-year post discharge from NICU: Social support, attachment and level of depressive symptoms. BMC Pregnancy Childbirth.

[17] Berry M.J., Saito-Benz M., Gray C., Dyson R.M., Dellabarca P., Ebmeier S., Foley D., Elder D.E., Richardson V.F. (2017). Outcomes of 23- and 24-weeks gestation infants in Wellington, New Zealand: A single centre experience. Sci. Rep..

[18] Koc E., Demirel N., Bas A.Y., Isik D.W., Hirfanoglu I.M., Tunc T., Sari F.N., Karastekin G., Ozdemir R., Altunhan H. (2019). Early neonatal outcomes of very-low-birth-weight infants in Turkey: A prospective multicenter study of the Turkish Neonatal Society. PLoS ONE.

[19] Stroustrup A., Bragg J.B., Spear E.A., Aguiar A., Zimmerman E., Isler J.R., Busgang S.A., Curtin P.C., Gennings C., Andra S.S. (2019). Cohort profile: The neonatal intensive care unit hospital exposures and long-term health (NICU-HEALTH) cohort, a prospective preterm birth cohort in New York City. BMJ Open.

[20] Ericson J., Flacking R., Hellström-Westas L., Eriksson M. (2016). Changes in the prevalence of breast feeding in preterm infants discharged from neonatal units: A register study over 10 years. BMJ Open.

[21] Dusing S.C., Tripathi T., Marcinowski E.C., Thacker L.R., Brown L.F., Hendricks-Muñoz K.D. (2018). Supporting play exploration and early developmental intervention versus usual care to enhance development outcomes during the transition from the neonatal intensive care unit to home: A pilot randomized controlled trial. BMC Pediatr..

[22] Pineda R., Heiny E., Nellis P., Dunsirn-Baillie S., Wallendorf M., Smith J. (2020). The baby bridge program: A sustainable program that can improve therapy service delivery for preterm infants following NICU discharge. PLoS ONE.

[23] Lee S.Y., Chau J.P.C., Choi K.C., Lo S.H.S. (2019). Feasibility of a guided participation discharge program for very preterm infants in a neonatal intensive care unit: A randomized controlled trial. BMC Pediatr..

[24] Yi-Ling C., Tzu-Ying L., Meei-Ling G., Kuan-Chia L. (2019). The effectiveness of an intervention program for fathers of hospitalized preterm infants on paternal support and attachment 1 month after discharge. J. Perinat. Neonatal Nurs..

[25] Kim N.H., Youn Y.A., Cho S.J., Hwang J.H., Kim E.K., Kim E.A., Lee S.M. (2018). The predictors for the non-compliance to follow-up among very low birth weight infants in the Korean neonatal network. PLoS ONE.

[26] Ravarian A., Vameghi R., Heidarzadeh M., Nariman S., Sagheb S., Nori F., Saeedershadi F., Norozi M., Vameghi R. (2018). Factors influencing the attendance of preterm infants to neonatal follow up and early intervention services following discharge from neonatal intensive care unit during first year of life in Iran. Iran J. Child Neurol..

[27] Arksey H., O’Malley L. (2005). Scoping studies: Towards a methodological framework. Int. J. Soc. Res. Methodol..

[28] Seo H.J., Kim S.Y. (2018). What is scoping review?. Health Technol. Assess..

[29] Peters M.D., Godfrey C.M., Khalil H., McInerney P., Parker D., Cassia B. (2015). Guidance for conducting systematic scoping reviews. JBI Evid. Implement..

[30] Seo H.J. (2020). The scoping review approach to synthesize nursing research evidence. Korean J. Adult Nurs..

[31] Munn Z., Peters M.D.J., Stern C., Tufanaru C., McArthur A., Aromataris E. (2018). Systematic review or scoping review? Guidance for authors when choosing between a systematic or scoping review approach. BMC Med. Res. Methodol..

[32] Kim J.S., Shin H.S. (2016). Development of the developmental support competency scale for nurses caring for preterm infants. J. Korean Acad. Nurs..

[33] Kaye S. (2016). Historical trends in neonatal nursing. J. Pedrinat. Neonat. Nurs..

[34] Hintz S.R., Gould J.B., Bennett M.V., Lu T., Gray E.E., Jocson M.A.L., Fuller M.G., Lee H.C. (2019). Factors associated with successful first high-risk infant clinic visit for very low birth weight infants in California. J. Pediatr..

[35] Swearingen C., Simpson P., Cabacungan E., Cohen S. (2020). Social disparities negatively impact neonatal follow-up clinic attendance of premature infants discharged from the neonatal intensive care unit. J. Perinatol..

[36] Feehan K., Kehinde F., Sachs K., Mossabeb R., Berhane Z., Pachter L.M., Broady S., Turchi R.M. (2020). Development of a multidisciplinary medical home program for NICU graduates. Matern. Child Health J..

[37] Liu Y., McGowan E., Tucker R., Glasgow L., Kluckman M., Vohr B. (2018). Transition home plus program reduces medicaid spending and health care use for high-risk infants admitted to the neonatal intensive care unit for 5 or more days. J. Pediatr..

[38] Whittingham K., Boyd R.N., Sanders M.R., Colditz P. (2014). Parenting and prematurity: Understanding parent experience and preferences for support. J. Child Fam. Stud..

[39] Brummelte S., Grunau R.E., Synnes A.R., Whitfield M.F., Thomas J.P. (2011). Declining cognitive development from 8 to 18 months in preterm children predicts persisting higher parenting stress. Early Hum. Dev..

[40] Suttora C., Spinelli M., Monzani D. (2014). From prematurity to parenting stress: The mediating role of perinatal post-traumatic stress disorder. Eur. J. Dev. Psychol..

[41] Jung S.Y., Tak Y.R. (2017). Family-centered care for hospitalized children: Concept analysis. Child Health Nur. Res..

[42] Yoo S.Y., Cho H. (2020). Exploring the influences of nurses’ partnership with parents, attitude to families’ importance in nursing care, and professional self-efficacy on quality of pediatric nursing care: A path model. Int. J. Environ. Res. Public Health.

[43] Castel S., Creveuil C., Beunard A., Blaizot X., Proia N., Guillois B. (2016). Effects of an intervention program on maternal and paternal parenting stress after preterm birth: A randomized trial. Early Hum. Dev..

[44] Koreska M., Petersen M., Andersen B.L., Brødsgaard A. (2020). Supporting families on their journey towards a normal everyday life facilitating partnership in an early discharge program for families with premature infants. J. Spec. Pediatr. Nurs..

[45] Hendricks-Munoz K.D., Prendergast C.C. (2007). Barriers to provision of developmental care in the neonatal intensive care unit: Neonatal nursing perceptions. Am. J. Perinatol..

[46] Luu T.M., Xie L.F., Peckre P., Cote S., Karsenti T., Walker C.D., Gosselin J. (2017). Web-based intervention to teach developmentally supportive care to parents of preterm infants: Feasibility and acceptability study. JMIR Res. Protoc..

[47] Majewska J., Zajkiewicz K., Wacław-Abdul K., Baran J., Szymczyk D. (2018). Neuromotor development of children aged 6 and 7 years born before the 30th week gestation. BioMed Res. Int..

