# Supplementary material for: Research Trends of Follow-Up Care after Neonatal Intensive Care Unit Graduation for Children Born Preterm: A Scoping Review
Source: Int J Environ Res Public Health. 2021 Mar 22;18(6):3268. doi: 10.3390/ijerph18063268 (PMC8004188; doi:10.3390/ijerph18063268)
Supplement: Supplementary file 1 [file ijerph-18-03268-s001.zip › ijerph-1151782-supplementary/Supplementary S1_0321.docx]

**Supplementary S1.** Database search strategies- Search Formula

| **Database** | **Search** | **Queries** | **Item Found** |
| --- | --- | --- | --- |
| Pubmed | #5 | ((((premature) OR (preterm)) AND ((((discharge) OR (graduate)) OR (follow up)) OR (follow-up))) AND (intensive care unit, neonatal [MeSH Terms])) AND ((education) OR (program)) | 681 |
|  | #4 | (education) OR (program) | 2,980,734 |
|  | #3 | intensive care unit, neonatal [MeSH Terms] | 31,118 |
|  | #2 | (((discharge) OR (graduate)) OR (follow up)) OR (follow-up) | 2,416,823 |
|  | #1 | (premature) OR (preterm) | 243,294 |
| CINAHL | S3 | S1 AND S2 | 107 |
|  | S2 | SU discharge OR SU graduate OR SU follow up AND TI discharge OR TI graduate OR TI follow up AND AB discharge OR AB graduate OR AB follow up | 6,496 |
|  | S1 | SU premature OR SU preterm OR SU preterm infants AND TI premature OR TI preterm OR TI preterm infants AND AB premature OR AB preterm OR AB preterm infants | 12,580 |
| Web of Science | #5 | #4 AND #3 | 200 |
|  | #4 | #2 OR #1 | 22,536 |
|  | #3 | TI=(education  OR  program) | 415,319 |
|  | #2 | TI=NICU | 2,115 |
|  | #1 | TI=(premature  OR  preterm  AND  (dischare OR graduate OR follow-up OR "follow up") | 20,485 |

Cumulative Index to Nursing and Allied Health Literature (CINAHL Plus with full text)

SU.EXACT = subject heading. TI,AB = terms in the title or abstract fields.
