# Supplementary material for: Research Trends of Follow-Up Care after Neonatal Intensive Care Unit Graduation for Children Born Preterm: A Scoping Review
Source: Int J Environ Res Public Health. 2021 Mar 22;18(6):3268. doi: 10.3390/ijerph18063268 (PMC8004188; doi:10.3390/ijerph18063268)
Supplement: Supplementary file 1 [file ijerph-18-03268-s001.zip › ijerph-1151782-supplementary/Supplementary S2_0321.docx]

**Supplementary S2.** A List of the Literature Reviewed for the Study

R1. Boykova, M. (2016). Transition from Hospital to home in preterm infants and their families. *J Perinat Neonatal Nurs, 30*(3), 270-272. doi:10.1097/jpn.0000000000000198

R2. Dusing, S. C., Tripathi, T., Marcinowski, E. C., Thacker, L. R., Brown, L. F., & Hendricks-Muñoz, K. D. (2018). Supporting play exploration and early developmental intervention versus usual care to enhance development outcomes during the transition from the neonatal intensive care unit to home: a pilot randomized controlled trial. *BMC Pediatr, 18*(1), 46. doi:10.1186/s12887-018-1011-4

R3. Ericson, J., Eriksson, M., Hellström-Westas, L., Hoddinott, P., & Flacking, R. (2018). Proactive telephone support provided to breastfeeding mothers of preterm infants after discharge: a randomised controlled trial. *Acta Paediatr, 107*(5), 791-798. doi:10.1111/apa.14257

R4. Feehan, K., Kehinde, F., Sachs, K., Mossabeb, R., Berhane, Z., Pachter, L. M., . . . Turchi, R. M. (2020). Development of a Multidisciplinary Medical Home Program for NICU Graduates. *Matern Child Health J, 24*(1), 11-21. doi:10.1007/s10995-019-02818-0

R5. Flores-Fenlon, N., Song, A. Y., Yeh, A., Gateau, K., Vanderbilt, D. L., Kipke, M., . . . Lakshmanan, A. (2019). Smartphones and text messaging are associated with higher parent quality of life scores and enrollment in early intervention after NICU discharge. *Clin Pediatr (Phila), 58*(8), 903-911. doi:10.1177/0009922819848080

R6. Goldstein, R. F., & Malcolm, W. F. (2019). Care of the neonatal intensive care unit graduate after discharge. *Pediatr Clin North Am, 66*(2), 489-508. doi:10.1016/j.pcl.2018.12.014

R7. Kuo, D. Z., Lyle, R. E., Casey, P. H., & Stille, C. J. (2017). Care system redesign for preterm children after discharge from the NICU. *Pediatrics, 139*(4), 1-8. doi:10.1542/peds.2016-2969

R8. Lipner, H. S., & Huron, R. F. (2018). Developmental and interprofessional care of the preterm Infant: neonatal intensive care unit through high-risk infant follow-up. *Pediatr Clin North Am, 65*(1), 135-141. doi:10.1016/j.pcl.2017.08.026

R9. Litt, J. S., Glymour, M. M., Hauser-Cram, P., Hehir, T., & McCormick, M. C. (2018). Early intervention services improve school-age functional outcome among neonatal intensive care unit graduates. *Acad Pediatr, 18*(4), 468-474. doi:10.1016/j.acap.2017.07.011

R10. Liu, Y., McGowan, E., Tucker, R., Glasgow, L., Kluckman, M., & Vohr, B. (2018). Transition home plus program reduces medicaid spending and health care use for high-risk infants admitted to the neonatal intensive care unit for 5 or more days. *J Pediatr, 200*, 91-97.e3. doi:10.1016/j.jpeds.2018.04.038

R11. Nayak, B. S., Lewis, L. E., Margaret, B., Bhat, Y. R., D'Almeida, J., & Phagdol, T. (2019). Randomized controlled trial on effectiveness of mHealth (mobile/smartphone) based Preterm Home Care Program on developmental outcomes of preterms: Study protocol. *J Adv Nurs, 75*(2), 452-460. doi:10.1111/jan.13879

R12. Pineda, R., Heiny, E., Nellis, P., Smith, J., McGrath, J. M., Collins, M., & Barker, A. (2020). The Baby Bridge program: A sustainable program that can improve therapy service delivery for preterm infants following NICU discharge. *Plos One, 15*(5), e0233411. doi:10.1371/journal.pone.0233411

R13. Robinson, C., Gund, A., Sjöqvist, B. A., & Bry, K. (2016). Using telemedicine in the care of newborn infants after discharge from a neonatal intensive care unit reduced the need of hospital visits. *Acta Paediatr, 105*(8), 902-909. doi:10.1111/apa.13407

R14. Toral-López, I., González-Carrión, M. P., Rivas-Campos, A., Lafuente-Lorca, J., Castillo-Vera, J., de Casas, C., & Peña-Caballero, M. (2017). Evolution of care indicators after an early discharge intervention in preterm infants. *Enferm Clin, 27*(4), 235-240. doi:10.1016/j.enfcli.2017.05.002

R15. Vohr, B., McGowan, E., Keszler, L., O'Donnell, M., Hawes, K., & Tucker, R. (2018). Effects of a transition home program on preterm infant emergency room visits within 90 days of discharge. *Journal of Perinatology, 38*(2), 185-190. doi:10.1038/jp.2017.136
