# Supplementary material for: Research Trends of Follow-Up Care after Neonatal Intensive Care Unit Graduation for Children Born Preterm: A Scoping Review
Source: Int J Environ Res Public Health. 2021 Mar 22;18(6):3268. doi: 10.3390/ijerph18063268 (PMC8004188; doi:10.3390/ijerph18063268)
Supplement: Supplementary file 1 [file ijerph-18-03268-s001.zip › ijerph-1151782-supplementary/Supplementary S3_0321.docx]

**Supplementary S3.** Summary of the Reviewed Researches (N=15).

| **No.** | **1st Author  (year)** | **Academic  Field** | **Research  Nation** | **Study Design** | **Subject (Sample size)** | **Study Aim** | **Key Finding** | **Elements of Follow-Up Care** |
| --- | --- | --- | --- | --- | --- | --- | --- | --- |
| 1 | Boykova (2016) | Nursing | USA | Literature review | Reports (50) | To explore a clear conceptualization of particular transition coupled with the challenges parents have when they return home and higher costs of healthcare service usage post-discharge | Five themes emerged from the review:  (1) disruption of parental role development, (2) distorted development of parent-infant relationships, (3) psychological consequences of a preterm birth and infant hospitalization, (4) learning caregiving and parenting, (5) need for social and professional supports which appear to reflect parental challenges during transition from hospital to home after discharge. | - Parental role development  - Development of parent-infant relationships  - Psychological consequences of a preterm birth and infant hospitalization  - Learning caregiving and parenting  - Need for social and professional supports which appear to reflect parental challenges |
| 2 | Dusing  (2018) | Physical therapy | USA | Single blinded randomized  pilot clinical  trial experimental study | Infants born very  preterm  or neonatal  brain injury  (EG:7, CG: 7) | To evaluate the initial efficacy of SPEEDI to improve early reaching and exploratory problem-solving behaviors. | . SPEEDI group enhanced early exploratory problem-solving behaviors | . Support the infants emerging  - motor development  - cognitive development |
| 3 | Ericson  (2018) | Nursing | Sweden | Randomized  controlled trial with stratified blocks experimental study | Mothers of preterm infants  (EG:231, CG:262) | To evaluate the effectiveness of proactive telephone support provided to breastfeeding mothers of preterm infants after discharge from NICU. | . The proactive telephone support was not associated with exclusive breastfeeding, breastfeeding satisfaction, attachment, and quality of life.  . Intervention group mothers experienced less parental stress in the dimensions of role restriction | - Person centered care  - Support for breastfeeding |
| 4 | Feehan  (2020) | Medicine | USA | Descriptive  study | Cohort of patients in the Next Steps Program  (549) | To discusses a multidisciplinary,  family-centered medical home designed to  address the needs of infants discharged  from NICU | . Hospital utilization among this patient in the program cohort is trending down  . A multidisciplinary primary care medical home is a successful  model of patient care demonstrating favorable associations with health care utilization, care coordination, and addressing/improving family functioning and their experience. | - Continuity of care from the NICU to primary care  - Routine developmental surveillance  - Care coordination  - Proactive screening to address medical and social needs |
| 5 | Flores-Fenlon  (2019) | Medicine | USA | Cross-sectional study | One parent  of preterm infant  (169) | To evaluate the impact of access to communication technology on caregivers’ quality of life, neurodevelopmental, and medical outcomes in preterm infants | . Evaluating smartphone and email access (or text messaging) on NICU discharge is important when considering enrolment in community programs and caregiver’s quality of life. | Not applicable |
| 6 | Goldstein  (2019) | Medicine | USA | Review | Not applicable | To examine the most common post-discharge medical problems that may be present in former premature  and critically ill term infants and inform the PCP about expected outcomes and possible new problems that may be encountered. | . At discharge, NICU graduates may be dependent on technology such as supplemental oxygen, tracheostomy, mechanical ventilation, surgically placed feeding tube and feeding pump, and several types of monitors.  . Primary care physicians must have special knowledge and understanding of the medical complications of NICU graduates to coordinate their post-discharge care and provide then with an effective medical home. | - Prescribed medication  - Feeding, breastfeeding  - Catch-up growth  - Nutrition: achieving energy, protein, and mineral needs  - Subspecialty clinic referrals: neurologic problems (malformations of the CNS, ischemic brain injury, hemorrhagic brain injury, other neurologic problems), muscle tone abnormalities, CP, sensory impairment (vision, hearing), developmental delay  - Technology dependence: oxygen, apnea monitor, tracheostomy and home ventilator, feeding tube, home nursing service |
| 7 | Kuo  (2017) | Medicine | USA | Review | Not applicable | To discuss the rationale for NICU graduates as a priority population for health system redesign | . Promotion of health and wellness  for children born preterm who are discharged to the community setting entails population health management from the patient-centered medical home; management, clinical care protocols, and clinical support from the tertiary care-based center; and a favorable payer strategy that emphasizes support for chronic care management. | Not applicable |
| 8 | Lipner  (2018) | Medicine | USA | Literature review | Papers  (do not report) | Not report | . Proactive developmental monitoring and implementation of timely therapeutic and educational early intervention services are essential to continue to support optimal outcomes for preterm  infants. | - Developmental monitoring  - Gross/fine motor, cognitive/linguistic, behavior/social interaction  - Referrals for necessary therapeutic interventions  - Home carryover activities  - Financial resources  - Parents' mental health |
| 9 | Litt  (2018) | Medicine | USA | Retrospective cohort study | Kindergartener born preterm (405) | To evaluate the effect of community-based EI services on the functional outcomes of high-risk  infants at school age | . Prompt enrolment in EI after recognition of an issue and greater breadth and intensity of services have a significant and positive effect on functional outcomes in kindergarten.  . Longer, more intense services were associated with higher kindergarten skills ratings in children at risk for disabilities. | Not applicable |
| 10 | Liu  (2018) | Medicine | USA | Control group,  pre-posttest quasi-experimental study | Infants cared for in the NICU  (EG=321,  CG=365) | To evaluate the effects of a transition home intervention on total Medicaid spending, emergency department visits, and unplanned readmissions for preterm infants born at ≤36 6/7 weeks gestation and high-risk full-term infants | . Transition home support services for high-risk infants provided both in the NICU and for 90 days after discharge by social workers and family resource specialists working with the medical team can reduce Medicaid spending and health care use. | - An interdisciplinary team of physicians, nurse practitioners, social workers, and family resource specialists  - Individualized family-centered  - Culturally sensitive support  - Provide education  - Link the family to appropriate community resources |
| 11 | Nayak  (2019) | Nursing | India | prospective, randomized controlled clinical trial  Experimental study | Infant born preterm  (EG:150,  CG: 150) | To evaluate the effectiveness of mHealthPHCP | . Empowering mothers and community health workers by integrating mobile technology into health care can help promote  healthy preterms, enhance developmental outcomes and  follow up, which in turn can reduce the mortalities, morbidities, and disabilities associated with prematurity. | - Parent‐infant interaction  - Remote monitoring of preterms growth parameters, developmental milestones  - Tracking immunization status  - Facilitating early recognition of danger signs  - Monitoring the breastfeeding  - Complementary feeding practice  - Hygiene practices including bath, massage, and diaper care  - Stimulating activities and toy safety |
| 12 | Pineda  (2020) | Occupational therapy | USA | Prospective descriptive study | High-risk infants after NICU discharged  (95) | To determine revenues and costs over time to assess the sustainability of the Baby Bridge program. | . There were initial losses during phase-in of Baby Bridge programming associated with operating far below capacity, yet the program achieved sustainability within 16 months of implementation | - Occupational therapy |
| 13 | Robinson  (2016) | Medicine | Sweden | randomized controlled trial, Control group, pre-posttest experimental study | Families with infants discharged NICU  (EG: 47, CG: 42) | To investigate whether the combined use of a web application and video calls would improve the comfort level of parents taking care of their infants at home and decrease the need for scheduled or emergency visits to the hospital. | . The use of the web page and video calls decreased the number of emergency visits to the hospital  . The parents were highly satisfied with the use of telemedicine (web app) | - Check of weight, length, and head circumference  - Parents' questions and concerns  - How to care infant |
| 14 | Toral-López  (2017) | Unknown | Spain | Control group, pre-posttest quasi-experimental study | Preterm infant admitted in NICU  (EG=46, CG=40) | To evaluate the evolution of health outcomes in preterm infants included in an early discharge program. | . The early discharge of preterm infants followed up at home by an expert nurse in neonatal care is a health service that achieves results in preparing parents for the care  of their child, enabling them to learn about the health services, adapt to their new life, and establish breastfeeding times. | Not applicable |
| 15 | Vohr  (2018) | Medicine | USA | Prospective cohort study | Mothers (804)  Preterm infant  (954) | To evaluate the effects of a THP and risk factors on ER use within 90 days of discharge | . Enhanced THP services were associated with a 33% decreased risk of all ER visits by year 3.  . Social and environmental risk factors contribute to preventable ER visits. | Not applicable |

CG=Control Group; CNS= Central Nervous System; CP= Cerebral Palsy; EG= Experimental Group; EI= Early Intervention; ER= Emergency Room; mHealthPHCP= mobile health based Preterm Home Care Program; NICU= Neonatal Intensive Care Unit; PCP= Primary Care Provider; SPEEDIE= Supporting Play Exploration and Early Development Intervention; THP= Transition Home Program;
